# Gamma-secretase modulators: a promising route for the treatment of Alzheimer's disease

**DOI:** 10.3389/fnmol.2023.1279740

**Published:** 2023-10-16

**Authors:** Gunnar Nordvall, Johan Lundkvist, Johan Sandin

**Affiliations:** ^1^AlzeCure Pharma AB, Huddinge, Sweden; ^2^Department of Neurobiology, Care Sciences, and Society, Division of Neurogeriatrics, Center for Alzheimer Research, Karolinska Institutet, Stockholm, Sweden; ^3^Sinfonia Biotherapeutics AB, Huddinge, Sweden

**Keywords:** gamma-secretase, gamma-secretase modulator, GSM, Alzheimer's disease, APP, amyloid-beta

## Abstract

Recent clinical data with three therapeutic anti-Aβ antibodies have demonstrated that removal of Aβ-amyloid plaques in early Alzheimer's disease (AD) can attenuate disease progression. This ground-breaking progress in AD medicine has validated both the amyloid cascade hypothesis and Aβ-amyloid as therapeutic targets. These results also strongly support therapeutic approaches that aim to reduce the production of amyloidogenic Aβ to prevent the formation of Aβ-pathology. One such strategy, so-called gamma-secretase modulators (GSM), has been thoroughly explored in preclinical settings but has yet to be fully tested in clinical trials. Recent scientific progress has shed new light on the role of Aβ in Alzheimer's disease and suggests that GSMs exhibit specific pharmacological features that hold great promise for the prevention and treatment of Alzheimer's disease. In this short review, we discuss the data that support why it is important to continue to progress in this class of compounds.

## Introduction

Alzheimer's disease (AD) is the most common form of dementia, affecting millions of people worldwide. AD therapies have recently been limited to symptomatic treatments, with Memantine representing the latest approved treatment for symptoms ~20 years ago (Witt et al., 2004). However, since 2021, the AD field has experienced a rebirth (Vellas and Aisen, 2021), highlighted by positive clinical data with three monoclonal antibodies (mAbs), Aducunumab (Haeberlein et al., 2022), Lecanemab (Dyck et al., 2022), and Donanemab (Sims et al., 2023).

In large phase 3 trials, these mAbs targeted the amyloid-beta (Aβ) component of AD and demonstrated Aβ amyloid clearance, along with significant disease-modifying effects in early AD. Together, these studies have proven the Aβ amyloid-cascade hypothesis in AD and shown that the course of AD can be treated therapeutically. Unfortunately, in a subset of patients, these therapies cause amyloid-related image abnormalities (ARIA), such as micro-hemorrhages (ARIA-H) and oedemas (ARIA-E), which is an important safety concern.

Encouraged by the progress, current drug discovery efforts steer toward more effective and safe treatments that ultimately could prevent Aβ amyloidogenesis and AD. This could be achieved with small-molecule treatments, providing cost-effective, patient/user-friendly oral therapies that would be fit for purpose as a chronic preventive treatment paradigm in people with emerging amyloidosis who are otherwise unaffected by the disease.

Extracellular Aβ-amyloid plaques, so-called “senile plaques”, are key neuropathological hallmarks of AD, originally described by Alois Alzheimer (Alzheimer, 1907). These extracellular proteinaceous deposits contain aggregates of the amyloid-beta peptide (Glenner and Wong, 1984; Surguchov et al., 2023). Seminal genetic discoveries in the 1990s linked early-onset familial AD (FAD) to three genes: the amyloid precursor protein (APP) and the presenilin (PS) 1 and 2 encoding genes (Bagyinszky et al., 2014). These genes were soon demonstrated to be directly involved in Aβ generation and accelerate the development of Aβ-amyloid pathology, indicating a pivotal role for Aβ in AD pathogenesis. To date, more than 200 disease-causing mutations in the *APP* and *PS* genes have been identified (see https://www.alzforum.org/mutations). Owing to the development of sensitive biochemical and imaging biomarker technologies, it is possible to monitor the process of Aβ amyloidosis during disease progression. It appears that the process of Aβ-amyloidosis begins ~10–15 years prior to the onset of symptoms in both sporadic AD and FAD. These findings could be viewed as optimistic since they provide opportunities to detect and treat AD early, many years before the overt symptomatic phase of the disease.

**Figure 1:**
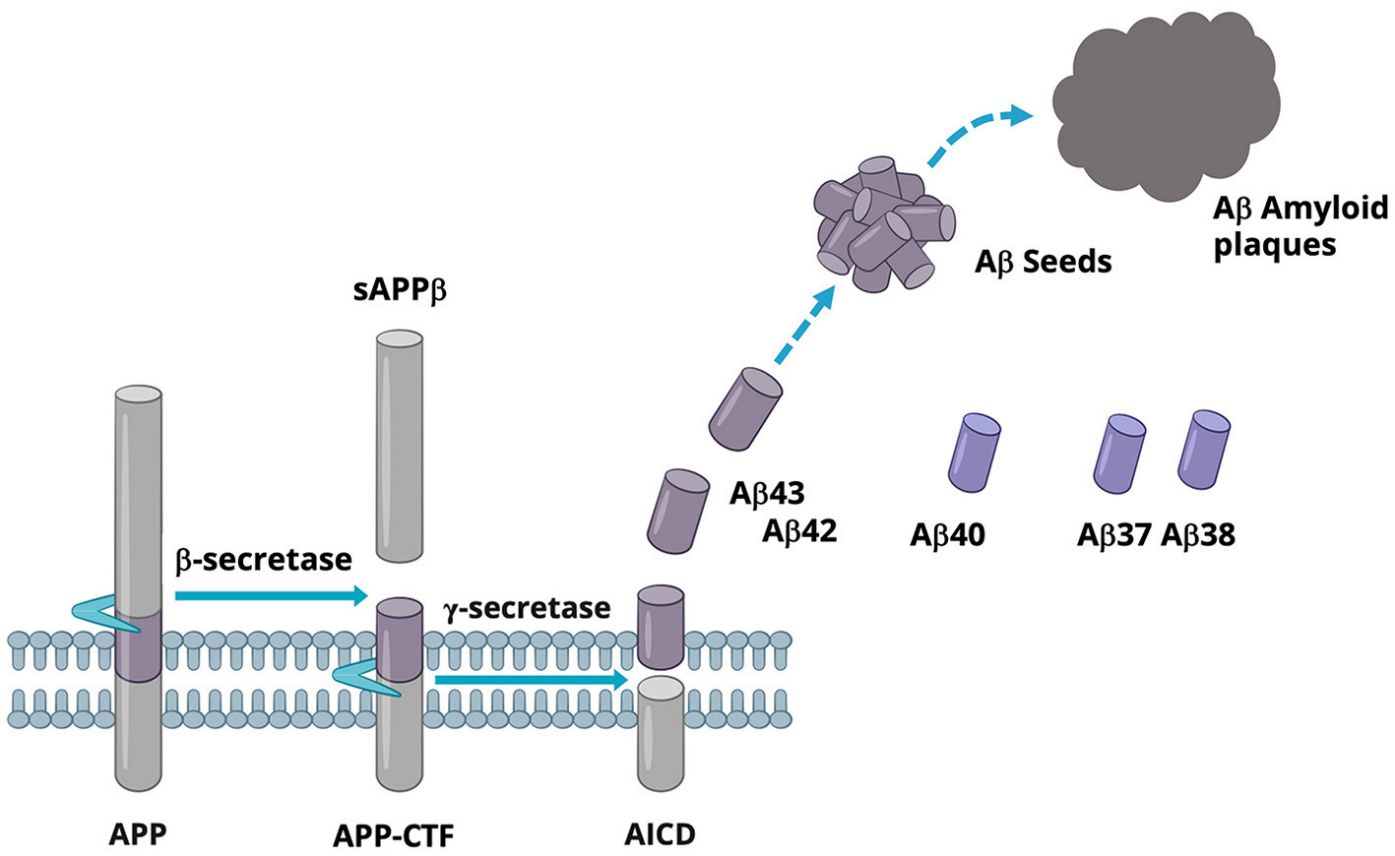
A schematic view of Aβ production.

Aβ is a family of secreted peptides generated from the sequential cleavages of the type 1 membrane protein APP by beta-secretase (BACE) and gamma-secretase (GSEC), respectively. BACE cleaves APP in the luminal domain, releasing the N-terminal soluble APPβ domain and leaving the C-terminal fragment, APP-CTF, which remains in the membrane. Subsequently, the APP-CTF is recruited to GSEC, a complex comprising four subunits, including PS, which harbors the active site. GSEC first cuts APP-CTF at the epsilon-cleavage site located close to the inner leaflet of the membrane. This cleavage event produces either Aβ48 or Aβ49 and the APP intracellular domain (AICD). The membrane-retained Aβ48 or Aβ49 is then further processed by GSEC in a continuous cascade of proteolytical events at every third of fourth amino acid, where the N-terminal product of each reaction becomes the substrate for the next GSEC cleavage event. Accordingly, GSEC processes APP-CTF along two main product lines, Aβ49 → 46 → 43 → 40 → 37… and Aβ48 → 45 → 42 → 38…, respectively (Takami et al., 2009; Matsumura et al., 2014; Olsson et al., 2014). During this processing cascade, Aβ43 and shorter Aβ peptides stochastically escape further processing by GSEC and are released into the extracellular space. As a result, Aβ peptides varying from 30 to 43 amino acids in length are secreted into the extracellular space. Among all secreted Aβ, Aβ40 is the most abundant in human CSF, followed by Aβ38, Aβ42, and Aβ37 (Liu et al., 2022). In cognitively normal individuals, Aβ42 and Aβ43 represent a smaller portion of the total secreted Aβ (Liu et al., 2022). These longer forms of Aβ seed the formation of Aβ-amyloid aggregates, a key step in the formation of amyloid plaques (Veugelen et al., 2016), as illustrated in Figure 1. Aβ42, which is produced in higher amounts than Aβ43, is the most abundant Aβ in amyloid plaques (Welander et al., 2009).

In FAD, the disease-causing mutations in PS appear to shift the Aβ product formation toward longer, more amyloidogenic forms of Aβ at the expense of shorter forms of Aβ (Sun et al., 2017). The preference of the FAD mutants for the generation of the more aggregation-prone Aβ42 and Aβ43 has been shown to be due to a destabilization of GSEC and incomplete processing through the Aβ product lines (Chávez-Gutiérrez et al., 2012; Szaruga et al., 2017). Interestingly, some PS1 mutations show an unaltered or only marginal increase in Aβ42/43 but are accompanied by a significant reduction in shorter Aβ, resulting in a decrease in total Aβ produced (Bentahir et al., 2006). But still, these Aβ profiles lead to accelerated amyloidosis, implicating that not only are the absolute levels of amyloidogenic Aβ critical for amyloidosis but also a decrease in shorter forms of Aβ may promote Aβ-amyloidosis.

Indeed, a growing body of scientific data support the pathogenic significance of an altered ratio between the shorter and longer forms of Aβ in AD. Liu et al. (2022) made the important observation that the actual ratio between Aβ37 or Aβ38 and Aβ42 or Aβ43 in human CSF correlates with the age of onset and the Mini-Mental State Exam (MMSE) in a range of FAD mutant carriers. Similarly, Petit et al. (2022a) found a correlation between the age of onset in familial AD caused by different PS1 mutants and the ratio of short peptides (Aβ37 + 38 + 40) over long peptides (Aβ42 + 43) produced, as studied in cell culture models. Indeed, the higher the (Aβ42 + 43)/(Aβ37 + 38 + 40) ratio, the earlier the age of onset of disease. Congruent with these findings, Cullen et al. (2022) have recently demonstrated a positive correlation between CSF Aβ38 levels and protection from developing AD. Although the mechanism by which shorter Aβ affects Aβ-amyloidosis is not fully understood, several studies have demonstrated that the shorter peptides Aβ37, Aβ38, and Aβ40 can individually and cooperatively inhibit the aggregation of Aβ42 (Kim et al., 2007; Moore et al., 2018; Nordvall et al., 2018; Braun et al., 2022), supporting an anti-amyloidogenic role of short forms of Aβ. Collectively, these data reveal the key importance of functional GSEC and complete Aβ product processing in maintaining a low Aβ long/Aβ short ratio, which could prevent the development of AD.

The pivotal role of Aβ-amyloid in AD has, for the last 25 years, engaged an enormous amount of drug discovery efforts targeting existing Aβ pathology, Aβ clearance, and Aβ production.

For the scope of this review, we will focus on the major therapeutic strategies explored to date to reduce Aβ production, with a special emphasis on GSMs.

Several approaches to reducing Aβ production have been explored in clinical studies. Inhibitors of gamma-secretase (GSIs) produced robust Aβ lowering in animals and were tested in clinical trials (Semagacestat Phase 3, Avagacestat Phase 2) (Doody et al., 2013; Coric et al., 2015). However, the inhibition of GSEC was associated with severe side effects, including cognitive worsening. These side effects were mainly mechanism-related due to the inhibition of other GSEC-dependent signaling events. More than 150 different GSEC substrates have so far been identified (Güner and Lichtenthaler, 2020). Many of these substrates, including the Notch family of receptors, mediate pivotal signaling both during development and in adults, and many GSI-associated side effects have indeed been linked to disturbed Notch signaling (Milano et al., 2004).

Another approach to reduce Aβ production that was tested in the clinic was to inhibit beta-secretase (BACE1). Five different BACE1 inhibitors were tested in late-stage clinical testing in mild-to-moderate AD, prodromal AD, and in people at risk of developing AD (for review see Imbimbo and Watling, 2019). Despite a large reduction in CSF Aβ42 levels, these compounds failed to show clinical benefit or were terminated due to futility and, somewhat unexpectedly, impaired cognitive abilities in the patients (Wessels et al., 2020). The explanation for these side effects is still a matter of debate. Clearly, like GSEC, BACE1 plays a pivotal role in neurobiology and has more than 40 substrates, some of which are involved in various synaptic functions like axonal guidance, neuronal plasticity, and LTP such as seizure protein 6 (SEZ6), CHL1, and neuregulin-1 (Munro et al., 2016; Yan, 2017; Müller et al., 2023). Therefore, it is likely that the safety liabilities discovered in the clinic with BACE1 inhibitors are mechanism-related.

## Gamma-secretase modulators

In light of the unsuccessful clinical outcomes of GSIs and BACE1 inhibitors, the alternative way to reduce Aβ production using gamma-secretase modulators needs to be further assessed clinically. In 2001, a seminal article by Weggen et al. described the first GSMs as an alternative mechanism to modulate gamma-secretase-mediated Aβ production. It was discovered that certain non-steroidal anti-inflammatory drugs (NSAIDs, e.g., Ibuprofen, Indomethacin, and Sulindac sulfide) could modulate GSEC to lower the production of Aβ42 and concomitantly increase Aβ38 without affecting the total amount of Aβ (Weggen et al., 2001; Eriksen et al., 2003).

These encouraging findings led to clinical phase 3 trials in AD patients using the (*R*)-enantiomer of the NSAID flurbiprofen (Tarenflurbil, Flurizan^®^ from Myriad) (Green et al., 2009). However, this compound was unable to demonstrate effects on cognitive function, likely due to its very low potency (*in vitro* IC50 in high μM) and poor CNS penetration (Wan et al., 2009). Another NSAID derivative that was clinically tested was the GSM Itanapraced (CHF5074) from Chiesi Farmaceutici, which was tested in a phase 1 study but did not affect CSF Aβ42 levels (Ross et al., 2012), most likely due to its low potency. Several carboxylic acid derivatives with improved potency and physicochemical profiles were later developed (Peng et al., 2011; Rogers et al., 2012) but were never clinically tested. In parallel, attempts were made to identify new types of gamma-secretase modulators, first identified by Neurogenetics (Cheng et al., 2004), but several other pharmaceutical companies followed and identified non-carboxylic acid series of compounds, with most molecules containing an aryl-imidazole moiety—the “second generation” GSMs (Xia, 2019; Mekala et al., 2020; Wolfe, 2021; Hur, 2022; Luo and Li, 2022). Recently, the structure of the gamma-secretase complex co-crystallized with the second-generation GSM E2012 developed by Eisai was determined (Yang et al., 2021). An *in silico* model supported by mutational data suggests that imidazole-based GSMs interact at the interface between GSEC and APP-C99, potentially providing new opportunities for drug design (Petit et al., 2022b). A similar structure has not been determined for the carboxylic acid class of GSMs, and how this class of GSMs modulates Aβ remains obscure. The binding sites for the two classes of GSMs are likely different since they do not show competitive binding, and they affect the processing of APP differently, resulting in different Aβ profiles (Borgegård et al., 2012; Olsson et al., 2014). There is evidence suggesting that the carboxylic acid class of GSMs interacts with APP rather than GSEC (Kukar et al., 2008), a finding coherent with the fact that both classes of GSMs have synergistic properties in reducing longer forms of Aβ (Robertson et al., 2017; Luo et al., 2022).

No GSMs of the “second generation” have reached phase 2 clinical trials yet, but several have demonstrated impressive activity in preclinical studies (Kounnas et al., 2010; Wanngren et al., 2012; Toyn et al., 2014, 2016; Brendel et al., 2015; Ratni et al., 2020; Rynearson et al., 2021), and some have been tested in phase 1 clinical trials. The first (non-NSAID) GSM tested in human phase 1 trials was E2012, which produced a ~50% reduction of plasma Aβ42 (Nagy et al., 2010). However, this compound showed some unacceptable side effects by affecting cholesterol metabolism, leading to lenticular opacity (Nakano-Ito et al., 2014). This side-effect was absent in phase 1 trials with the follow-up compound E2212, which robustly lowered plasma Aβ42 and did not display any serious adverse events (Yu et al., 2014). Still, E2212 was not further developed for undisclosed reasons. BMS demonstrated that their GSM BMS-932481 produced a large increase in Aβ37 and a reduction of Aβ42 CSF levels in healthy volunteers (Soares et al., 2016). However, compound-related adverse liver findings were seen after repeated dosing (Zhuo et al., 2023), which led to the termination of further studies. Neurogenetics performed a small phase 1 study with NGP 555 (Kounnas et al., 2017), which increased the Aβ37/Aβ42 ratio and appeared well tolerated, but no further development has been reported. Pfizer showed promising phase 1 SAD and MAD data with PF-06648671 demonstrating reductions of Aβ42 and Aβ40, together with increases in Aβ37 in healthy volunteers (Ahn et al., 2019). No major side effects were reported, but the compound did not progress further, potentially due to Pfizer's decision to leave the CNS therapeutic area. Currently, only a limited number of GSM programs appear to be active, including UCSD-776890 from the group of Steven Wagner that received NIH funding for a Phase 1 study (Rynearson et al., 2021). Roche has recently completed a phase 1 study with their GSM RG6289 (Ratni et al., 2020; Sturm et al., 2023), and AlzeCure Pharma is developing GSMs within their Alzstatin platform (Sandin et al., 2022). The key requirements for an effective GSM are high potency, good CNS exposure, and PK properties to provide robust Aβ42 reductions at reasonable doses. The safety of the compound is of paramount importance, as many GSMs have suffered from insufficient margins between efficient Aβ42-lowering effects and compound-related side effects. This is probably a consequence of the binding site requiring compounds with high logP, flat structures with high aromatic content, and an imidazole moiety, potentially leading to poor selectivity. Recent examples have shown that it is indeed possible to develop GSMs with reduced aromaticity and planarity, as well as to avoid an imidazole moiety (Ratni et al., 2020).

The pharmacology of GSMs provides a number of key features that hold great promise as a preferred treatment to prevent amyloidogenic Aβ production. First, it appears to be a safe, tolerable mechanism. In contrast to GSIs and BACE inhibitors, GSMs do not inhibit any enzyme but rather *modulate* the activity of GSEC. Thus, neither Notch processing nor other important signaling pathways dependent on GSEC appear to be affected by GSMs (Weggen et al., 2001; Wanngren et al., 2012). Furthermore, several tested GSMs have been shown to be selective for APP processing and Aβ modulation, demonstrating that it is feasible to design GSMs tailored for Aβ modulation (Wanngren et al., 2012; Weber et al., 2021). These are critical attributes of GSMs and minimize the safety liabilities that have been associated with both BACE and GSEC inhibitors. In fact, currently, no mechanism-related toxicity has been assigned to GSMs, which is promising considering their potential use as an early, preventive, chronic treatment in individuals at risk of developing AD.

**Figure 2:**
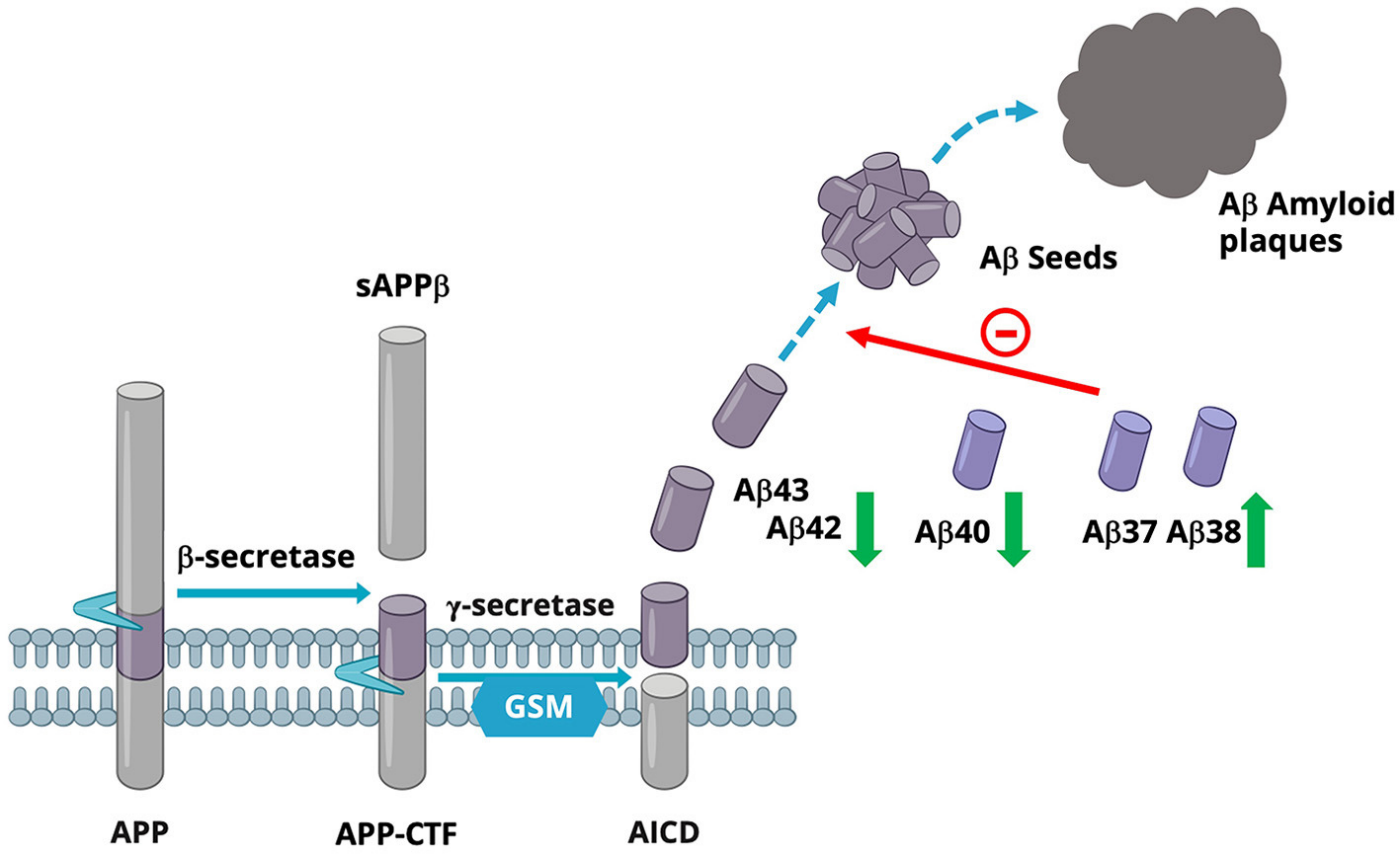
Schematic view of Aβ production in the presence of a GSM.

Second, GSMs are effective anti-amyloidogenic agents. GSMs do not change the total amount of Aβ formed but rather decrease the production of amyloidogenic Aβ while increasing the production of shorter Aβ. This is the *opposite effect* on Aβ generation as compared to the situation with several FAD-causing mutations in the *PS* genes (which accelerate amyloidosis). Indeed, GSMs reduce the production of the aggregation-prone Aβ42 and most likely Aβ43, as well as Aβ40 (Olsson et al., 2014), while the levels of Aβ37 and Aβ38 are increased. Studies have shown that GSMs appear to stabilize the GSEC/APP-CTF complex, allowing GSEC to continue processing Aβ43/Aβ42/Aβ40 into the shorter forms Aβ37 and Aβ38, thus increasing the turnover of the longer forms of Aβ (Olsson et al., 2014; Szaruga et al., 2017). Interestingly, the increase of the shorter Aβ37 and Aβ38 could have several beneficial effects, including attenuation of Aβ42-mediated toxicity (Moore et al., 2018; Quartey et al., 2021) and/or reduced Aβ42 aggregation (Nordvall et al., 2018; Braun et al., 2022). These effects would lead to decreased formation of Aβ pathology, and would represent an inverted Aβ pattern to that observed in FAD; see Figure 2. Indeed, higher levels of Aβ38 have been shown to be associated with a lower risk of AD-related changes in clinical studies (Cullen et al., 2022).

Finally, an increasing body of data suggests that Aβ itself may play an important physiological role in normal cellular processes (Sturchio et al., 2022). Since GSMs do not affect the total amount of Aβ peptides produced but only alter the ratio between longer and shorter Aβ forms, the potential signaling function of Aβ in the presence of GSMs may not be disturbed.

In light of the recent progress with Aβ immunotherapies in the clinic, combined with our increased understanding of the pathogenic mechanisms resulting in amyloidosis and FAD, GSMs hold great promise as a novel anti-amyloidogenic therapy. Based on our current knowledge, a GSM is unlikely to produce any major mechanism-related side effects, and with a profile that is the reverse of the familial mutations in PS, a GSM would be an excellent choice for the primary prevention of Alzheimer's disease (Voytyuk et al., 2018). This could be the ultimate goal when diagnostic and prognostic biomarkers have evolved even further to efficiently select and monitor the target population, which could include risk groups such as APOE4-positive individuals (Leonenko et al., 2021). Such a treatment needs to start early, well before amyloid deposition in the brain is initiated. In this case, it is conceivable that an early treatment with a GSM would provide a superior anti-amyloidogenic effect.

A secondary prevention approach could also be considered with a GSM. An increase in Aβ pathology as detected by PET is the first pathological change in AD, which in turn appears to subsequently drive the tau pathology (Zhang et al., 2021). Therefore, using a GSM prior to the rapid increase in tau pathology driven by Aβ could serve as an alternative strategy (Karran and Strooper, 2022).

Clinical evaluation of either primary or secondary prevention would probably require extended clinical trials. Therefore, evaluating a GSM as a maintenance therapy after Aβ-clearance with an anti-Aβ antibody with the aim of preventing the buildup of new amyloid aggregates could be an attractive option as a first step. The antibody treatment aims to clear plaques until amyloid levels are no longer detectable in PET scans (~20 centiloid). Once this is achieved, the treatment is stopped. Therefore, these patients would be “reset” to an approximate common starting level with no or low levels of amyloid plaques, and the buildup of plaques would then commence again. A GSM treatment at this stage, reducing the production of aggregation-prone Aβ species, would be a clinically feasible and suitable treatment option to reduce the buildup of new plaques.

Alzheimer's disease is a complex disease to treat and prevent. Anti-Aβ antibodies have reinvigorated the field by showing significant clinical benefits with treatment. We believe that GSMs will be an essential addition to the treatment toolbox for Alzheimer's disease, and one that is likely necessary for its ultimate prevention.

## Author contributions

GN: Writing—original draft, Writing—review and editing. JL: Writing—original draft, Writing—review and editing. JS: Writing—original draft, Writing—review and editing.
